# Labor markets for health supply chain management in Rwanda: a qualitative study of stakeholder perspectives

**DOI:** 10.1186/s12913-023-10304-1

**Published:** 2023-12-07

**Authors:** Erin Meier, Andrew N. Brown, Bridget McHenry, Inès K. Gege Buki, Michael Egharevba, Joseph Kabatende

**Affiliations:** 1 IntraHealth International, 6340 Quadrangle Drive, Suite 200, Chapel Hill, NC 27517 USA; 2 GHSI-III: a Social Solutions International Contract for USAID, Washington, DC, USA; 3 USAID Global Health Supply Chain Program-Procurement Supply Management (GHSC-PSM) Project, KG 514 St, Kigali, Rwanda; 4 USAID Global Health Supply Chain Program-Procurement Supply Management (GHSC-PSM) Project, 1275 New Jersey Avenue SE, Suite 200, Washington, DC, 20003-5115 USA; 5 Rwanda Food and Drugs Authority, PO Box 1948, Kigali, Rwanda

**Keywords:** Health supply chain, Labor market, Human resources, Public health, Rwanda, Workforce, Demand, Supply, Competency

## Abstract

**Background:**

Effective supply chains for health products require an adequate, skilled workforce for supply chain management (SCM). Rwanda faces challenges in human resources for SCM, including limited capacity for SCM at different levels. Understanding of the factors influencing the supply of and demand for SCM professionals in Rwanda is necessary to ensure the labor market contains an adequate workforce. This study identifies the perspectives of key stakeholders in the supply chain management sector about the factors influencing the supply of and demand for SCM professionals.

**Methods:**

Data were collected in semi-structured group and one-on-one interviews with 39 key stakeholders involved in the supply chain management labor market between March and April 2019. Interviewees were categorized according to their role in the labor market as system actors, functional actors involved in the supply of SCM workers, and functional actors involved in the demand for SCM workers. Interviewees were asked open-ended questions about factors influencing the demand for and the supply of SCM workers, and recommendations for improvement. Interviews were analyzed thematically. One validation focus group was held and the results were also reviewed by the Ministry of Health in Rwanda.

**Results:**

Stakeholders agreed that skills mismatch between SCM workers’ skills and the competencies jobs require impacts the supply of workers. A lack of career structure for SCM, lack of professional definitions for health supply chain management, and SCM curricula that do not match the needs of the workplace contribute to this gap. The demand for SCM professionals is poorly defined in terms of the numbers of professionals needed and the skills workers require. Financial limitations hinder demand for health SCM professionals.

**Conclusions:**

This study adds to the understanding of factors influencing the SCM labor market in Rwanda by documenting perspectives from government ministries, professional organizations, universities, and employers from SCM organizations. Improving the SCM labor market in Rwanda and the availability of the skilled cadres required for the effective management of health supply chains in Rwanda requires a coordinated effort by the Ministry of Health in Rwanda, private SCM companies, professional associations, education sector, and policy makers.

**Supplementary Information:**

The online version contains supplementary material available at 10.1186/s12913-023-10304-1.

## Background

The global health community has committed to accelerate efforts to achieve universal health coverage (UHC) by 2030 to improve access to health services and to safe, effective, quality, and affordable essential medicines and vaccines [1]. Health supply chain management (SCM), which involves “the design, planning, execution, control, and monitoring of supply chain activities” [2] for supply chains responsible for health commodities in the public and private sector, serves as a key enabler to achieve UHC, by ensuring that a continuous supply of affordable medicines is available for increased service coverage [3]. Health supply chains are complicated systems that depend on a skilled workforce with distinct technical competencies to function effectively [4–6]. The health SCM workforce manages appropriate commodity selection, forecasting, procurement, storage, distribution, and use of health commodities [7]. This workforce includes pharmacists, supply chain managers, logisticians, data managers, warehouse, transport personnel, and others. An adequate supply of skilled health SCM workers is critical to a strong health system [8, 9]; however, countries experience deficiencies in the number of workers with the required competencies [8, 10, 11] and this essential workforce is often overlooked in both the private and public sectors. All too often, health SCM workers lack the appropriate skills and training, career path, or empowerment to effectively manage the supply chain [8, 10, 12–14]. A 2017 study by the World Bank found competency deficiencies and workforce shortages across the supply chain workforce, particularly in low- or middle- income countries (LMIC) [6]. Meanwhile, a systematic literature review of health supply chain research found that the existing literature neglected the critical areas of employee training and human resource practices [15]. Workforce issues can limit the ability of health supply chains to meet existing demands [16]. Poorly functioning health supply chains jeopardize the major investments in health supplies made by governments and partners and can impact the health goals of countries [17].

In Rwanda, limited capacity for SCM remains a challenge [13, 18] and issues persist in the availability of medicines in the public sector [19]. Meanwhile, demand for health SCM workers is expected to increase as the Government of Rwanda aims to achieve UHC and to grow Rwanda’s pharmaceutical manufacturing industry [20]. Supply chains providing non-pharmaceutical products also are expected to grow as Rwanda’s economy expands [20]. This will increase demand for SCM professionals in the private sector and other government institutions**,** placing increased pressure on the health SCM labor market, as many of the same SCM skillsets apply across sectors.

In this labor market, supply refers to the pool of SCM workers willing to work in Rwanda’s SCM industry; demand for SCM labor refers to the employers [21, 22]. Many factors influence whether the supply of SCM workers meets the demand. For example, supply is influenced by the capacity of and curriculum at educational institutions that produce workers, regulations that determine requirements for SCM professionals, and dynamics that influence how SCM workers move between organizations [21]. Emigration of local professionals abroad or immigration of foreign workers also affects supply [21]. Other contributing factors impact retention and performance, including working conditions, skills, motivation, and budget allocations [4].

Labor market analyses that explore factors influencing supply and demand of SCM workers in the public and private sectors are needed so that policy recommendations can address identified challenges and improve labor market operation. Identifying the education sector’s influence on supply is also critical [21].

### Literature review

Although some labor market analyses within the health sector have been completed, including in Rwanda [23], the limited studies [21, 24–29] that exist focus on the labor market for doctors, nurses, and clinical cadres. Most of these studies have identified challenges in achieving an adequate supply of competent workers. For example, studies of the clinical health workforce in India, Bangladesh, Kenya, and Lesotho [26, 27, 29, 30] revealed health worker density levels below international benchmarks. A recent health labor market analysis in Rwanda indicated shortages of nearly all categories of the health workforce, despite annual increases in the number of registered nurses, midwives, and pharmacists, due to insufficient production of workers to meet projected needs [23]. The issues revealed in these studies of the health workforce are also expected in a study of health SCM workers. Few descriptive studies have explored the national labor market conditions impacting the availability of health SCM workers [9]. Non-clinical cadres are underrepresented in research on education sector capacity and labor markets.

Studies of SCM graduates and entry-level workers (not specific to the health sector) in high-income countries have assessed gaps between industry demand for workers and workforce supply. A survey of managers’ perceptions of the knowledge and skill gaps in graduates from SCM undergraduate programs in Oman found that managers’ satisfaction level with entry-level logistics professionals’ knowledge and skills was below expectations [31]. Studies in the United Kingdom [32] and the United States [33] compared undergraduate and graduate-level SCM curricula to the job requirements published by employers. Both studies found gaps between the industry’s demand for SCM talent and the supply of graduates from higher education institutions. Additional file 1 includes a table summarizing past studies on the labor market in the health or SCM sector. The authors in these studies did not investigate the perspective of education institutions producing SCM graduates. In Rwanda, information on the education sector’s production of SCM skills for various sectors is limited, as is information on the private sector’s demand for labor and skills.

### SCM demand environment in Rwanda

In Rwanda, legislation requires that pharmacists manage supply chains for pharmaceutical products; thus, supply chains for health commodities are managed separately from those for non-health commodities. In the health sector, public and private supply chains exist. Bonded warehouses and many private pharmaceutical wholesalers operate. Ministry of Health (MOH) distributes pharmaceutical products to public health facilities. A faith-based organization, Le Bureau des Formations médicales agréées du Rwanda (BUFMAR), distributes pharmaceutical products to all faith-based health facilities [13]. These entities, including health facilities, have demand for SCM workers in various capacities.

Meanwhile, non-pharmaceutical commodities (e.g., construction, agricultural, manufacturing goods) move through separate supply chains for private and public sector use. At the intersection of supply chains for health and non-health commodities, third-party logistics companies, fourth-party logistics companies, and humanitarian organizations operate.

### SCM supply landscape in Rwanda

Public and private universities produce the supply of workers through a limited offering of SCM courses for various sectors. Table 1 lists undergraduate and postgraduate degrees with SCM components offered. Much of the SCM workforce doesn’t have formalized SCM education but have developed skills through on-the-job experience. Foreign SCM professionals also enter the supply of workers from abroad.

**Table 1:** Formalized degrees with SCM components offered in Rwanda

Institution	School	Degree	Presence of SCM Components
University of Rwanda	The Regional Centre of Excellence for Vaccine Immunization and Health Supply Chain Management (RCE-VIHSCM)	Master’s course on health SCM	Significant health SCM core
University of Rwanda	RCE-VIHSCM	SCM professional short courses	Short training courses on health SCM
University of Rwanda	School of Medicine and Pharmacy	Bachelor of Sciences (BSc) with Honors in Pharmacy	Provides introductory SCM competencies
University of Rwanda	School of Medicine and Pharmacy	Bachelor of Medicine and Surgery	No SCM components
University of Rwanda	School of Health Sciences	Masters in Hospital Management	Limited procurement and SCM education
University of Rwanda	School of Business	BSc in logistics and SCM^a^	Significant SCM core; mainly focused on non-health commodities
University of Kigali	School of Business and Management	BSc with Honors in Procurement and Supplies	Significant SCM core; mainly focused on non-health commodities
University of Kigali	School of Business and Management	Master of Science in Procurement	Significant SCM core; mainly focused on non-health commodities
University of Kigali	School of Business and Management	PhD in Business Administration^a^	Limited procurement and SCM education

Table Key:

^a^Degree was planned but not yet offered at time research was conducted

### SCM system actors in Rwanda

Multiple government ministries regulate areas affecting this workforce, including the Ministries of: Public Service and Labor, Trade and Industry, Education, and Health [34, 35]. Within MOH, the Department of Clinical and Public Health Services oversees the health supply chain and conducts national procurement and distribution of pharmaceuticals; Rwanda Food and Drugs Authority (Rwanda FDA) regulates pharmaceutical products [13]. Rwanda Development Board (RDB) and Rwanda Public Procurement Authority (RPPA) focus, respectively, on developing the private sector and overseeing public procurement [36, 37]. Health professional councils – including National Pharmacy Council, Rwanda National Council of Nurses and Midwives, and Allied Health Professionals Council – play key roles in determining education standards and registration for health professionals.

### Research objective

Although studies have explored the labor market for clinical health workers in multiple countries, there is a lack of research on the national labor market conditions impacting the availability of and demand for health SCM workers [9]. A deeper understanding of the labor market affecting the health SCM workforce is required as evidence to guide the comprehensive actions needed to catalyze and improve this market. This study aims to reveal the factors influencing supply of and demand for the health SCM workforce in Rwanda through key informant interviews with the main stakeholders in the SCM sector, including government, education sector, and private sector entities. This is the first such study of the labor market for the health supply chain workforce.

## Methods

The study was conducted from March to April 2019. The City of Kigali, Rwanda’s capital, was selected among the five provinces because it is the location of national-level government ministries (system actors), universities, and many private supply chain companies. A qualitative approach was used to gather information on the factors influencing the SCM labor market in Rwanda. The experience of the authors suggested the quantity and skills of available SCM workers in Rwanda did not match employers’ demand for workers, and a qualitative approach that engaged government ministries, supply stakeholders, and demand stakeholders was thought the best approach to understand these issues in detail.

The methodology was reviewed and approved the by the Rwanda National Ethics Committee of the MOH. We conducted semi-structured interviews, of approximately one-hour duration, and one focus group. The purpose of the interviews was to gather perspectives from key stakeholders on factors influencing supply and demand of health SCM workers. The purpose of the focus group was to validate summarized data from interviews and to identify any missing factors affecting supply and demand.

The research team agreed to use purposive sampling to ensure that relevant system actors and functional (supply and demand) actors had the opportunity to participate in the study. Using an existing mapping of system actors and organizations on the supply and demand sides of the health SCM labor market in Rwanda, researchers selected a purposive sample of mapped organizations. The final number of interviews of supply and demand actors was determined by budget and the Kigali-focus of SCM organizations. (As noted previously, SCM system actors and the most influential supply and demand actors are present in Kigali). Proposed organizations were sent an invitation letter via MOH to participate in the study. An in-person interview was scheduled, and participants completed the informed consent form to provide written informed consent. Interviews lasted approximately one hour in duration. Two interviewers were present to collect data at each interview.

### Research instrument

We conducted semi-structured interviews using an interview guide (Additional file 2), which was developed based on existing literature and study objectives and approved by the Rwanda National Ethics Committee. The People that Deliver (PtD) Human Resource for Supply Chain Management Theory of Change was used as a basis for interview questions around human resources (HR) systems [4].

Interviewers collected responses about interviewees’ demographics, perceptions of factors affecting the organization’s demand for SCM workers, and perceptions of the labor market’s supply of SCM workers (presented in this paper). In addition, information was collected on the organization’s staff profile, hiring practices, benefits, satisfaction with employee skill levels, retention rates, training practices, and procedures for supportive supervision and performance evaluation.

Interviews were conducted in English by two interviewers using the approved interview guide. Questions were sent to interviewees in advance. Interviews were not recorded. One data collector took notes of each interview to capture summaries of responses to each question and certain verbatim quotations, with the notes reviewed by the second data collector at the conclusion of the interview. Written informed consent was obtained before starting the interview.

### Focus groups

After each interview, all participants were invited to join a focus group. Ten participants attended one focus group, which was aimed at validating interview findings and was conducted over a two-hour period. The focus group consent form was issued to obtain written informed consent. Researchers presented the thematic results from the interviews to the focus group participants for discussion and verification to validate the data. Focus group was not recorded. One data collector took notes of the focus group discussion to capture the group’s agreed upon feedback and additions to factors influencing supply and demand of SCM workers.

### Data analysis

Written notes from each interview were reviewed by both researchers after the interview and one researcher tagged responses according to the theme of the response. Grounded theory process was applied to guide the analysis [38]. The researcher completed line-by-line coding of the response excerpts to first identify categories. Themes were formed by determining which categories were dominant in the data and were representative of participant responses. To test the themes, the researcher checked what was said about each theme from multiple participant viewpoints. Themes of a similar nature were then grouped under representative headings and all data from those categories were compared. A second researcher reviewed written notes of each response and the consolidated groupings. Disagreements were discussed until agreement was reached. The number of times the theme appeared was recorded. Consolidated results were presented to the focus group for validation.

Grounded theory process was also used to analyze feedback from focus group participants about each of the seeding statements presented. For each of the seeding statements, researchers asked participants to prioritize the top three key messages shared by all participants. The final top three messages for each topic were added to the existing analysis of themes and integrated into the results. Messages provided that were not aligned to the themes in the seeding statements were noted and considered in the results. The Rwanda MOH and all researchers reviewed the final analysis of all data.

### Ethical considerations

Research protocol was approved by the Rwanda National Ethics Committee of the MOH (IRB00001497 of IORG0001100). Written informed consent was obtained from each study participant for face-to-face interviews and focus group participation, using templates approved by the ethics committee.

## Results

### Participant characteristics

Interviews were conducted with 39 individuals working at 29 institutions that represent the main stakeholders. Table 2 lists the number of participants, their organizational affiliation, and study category. Participants included government ministries, professional organizations, universities, nongovernmental organizations (NGOs), and private sector companies involved in supply chain, logistics, and pharmaceuticals. Participants were categorized into three categories based on their labor market involvement: 1) system actors (*n* = 10), 2) functional actors generating supply of SCM workers (*n* = 3), and 3) functional actors with demand for SCM workers (*n* = 26). System actors operate at the national level with a holistic view of supply chain systems in health and other industries (e.g., government ministries and professional associations). Functional actors on the supply side work for institutions producing SCM workers (e.g., universities). Functional actors on the demand side work for institutions employing SCM professionals (e.g., private sector companies, NGOs). One focus group was conducted, where previously analyzed themes were presented for validation. Ten participants took part: system actors (*n* = 3), functional actors generating supply of SCM workers (*n* = 2), and functional actors with demand for SCM workers (*n* = 5).

**Table 2:** List of the organizations participating in the study

Organizational Affiliation	Study Category	Description	Total interviewees
Ministry of Health: Department of Clinical and Public Health Services	System Actor	Government ministry	1
Ministry of Health: Rwanda Food and Drugs Authority (Rwanda FDA)	System Actor	Government ministry	1
Ministry of Public Service and Labor	System Actor	Government ministry	3
Ministry of Trade and Industry (MINICOM)	System Actor	Government ministry	1
National Pharmacy Council	System Actor	Professional Association	2
Allied Health Professionals Council	System Actor	Professional Association	1
National Council for Nurses and Midwives	System Actor	Professional Association	1
University of Rwanda, School of Public Health, Regional Centre of Excellence for Vaccine Immunization and Health Supply Chain Management (RCE-VIHSCM)	Functional actor: supply	Educational Institution	1
University of Rwanda, College of Medicine and Health Sciences, School of Medicine & Pharmacy	Functional actor: supply	Educational Institution	1
University of Rwanda, College of Business and Economics, School of Business	Functional actor: supply	Educational Institution	1
Medical Procurement and Production Division (MPPD)^a^	Functional actor: demand	Public central medical store	1
Magasins généraux du Rwanda (MAGERWA)	Functional actor: demand	Public bonded warehouse	1
USAID Global Health Supply Chain Program–Procurement Supply Management Project (GHSC-PSM)	Functional actor: demand	Technical Partner	1
United Nations High Commissioner for Refugees (UNHCR)	Functional actor: demand	Humanitarian Organization	3
Eight private sector pharmaceutical wholesaler and distributor companies	Functional actor: demand	Private sector pharmaceutical wholesalers and distributors	10
Five private sector non-pharmaceutical wholesalers and product distributor companies	Functional actor: demand	Private sector non-pharmaceutical wholesalers and product distributors	8
Two private sector transport and logistics operator companies	Functional actor: demand	Private sector transport and logistics operators	2
Only organizational level data was routinely collected but where more detailed department level data was provided and where there was low risk of participant identification, this data was included			

^a^In the time since data collection occurred, MPPD has transformed into Rwanda Medical Supply Ltd, a private, government-owned central medical store company separate from the MOH, which reports to and is supervised by the MOH

Results are presented for the main interview questions focusing on supply of and demand for SCM workers, with themed subheadings noted in order of the frequency of the themes. The terms majority, some, and individual are used to denote the frequency of these themes across the participants interviewed. Particular quotations are included, for themes where succinct, illustrative, representative quotations were available, to illustrate certain themes and to elucidate informants’ perspectives. The perspective of system actors, supply functional actors, and demand functional actors are presented separately. The interrelationship of subthemes is also summarized.

Across participant categories, the results identified six themes affecting demand for workers: 1) workload, 2) financial issues, 3) the expanding local pharmaceutical industry, 4) regulation, 5) education, skills, and competencies, and 6) staff turnover. The results identified nine main themes affecting supply of workers: 1) education, skills, and competencies, 2) lack of career structure, 3) labor laws, 4) outsourcing and networking, 5) limited SCM pharmacy curriculum, 6) disconnect between SCM industry and education sector, 7) facility and staff capacity at educational institutions, 8) lack of awareness of SCM career and courses, and 9) cost of education.

### Perspectives from interviews with system actors

Ten participants from seven organizations categorized as system actors were interviewed (Table 2).

#### Perspective of system actors regarding the factors affecting demand for SCM workers

The results from system actors identified three themes affecting demand for SCM workers, including: 1) workload, 2) financial limitations, and 3) the expanding local pharmaceutical industry. Themes are listed in order of the frequency reported.

1. Workload

The majority of interviewees described high workload across the government-run health supply chain, as evidenced by 1) an annual increase in commodities required and procured, and 2) increases in the number of patients treated. Government’s focus on universal health coverage and expanding access to services are expected to increase future workload. At some health facilities, SCM workload may prevent clinical activities from occurring.

*“There is high workload at hospital, district, and central levels. For example, at a hospital, [there might be] one pharmacist, but [they are] so busy that they can only focus on SCM and are not able to do other clinical activities. At a district, [which is] supplying all the catchment area, there is not enough workforce. [They are] just getting by day-to-day.”*

(System Actor Respondent 3)

2. Financial limitations

Some interviewees described how the public service structure drives labor demand and available funding limits jobs. Obtaining approval for new SCM positions within the public sector is challenging in a fiscally constrained environment.

*“The [public service] structure drives demand. [This] structure should be in line with capacity needed to accommodate a number of staff in each field… [The] available funding limits jobs. [There are a] number of SCM staff in the structure but [it’s] limited due to limited financial capacity. [The number is] not sufficient but staff are committed.”*

(System Actor Respondent 3)

3.  Expanding local pharmaceutical industry

Some interviewees noted that the MOH and MINICOM aim to expand the local pharmaceutical manufacturing market and establish multiple pharmaceutical manufacturers, which will increase demand for health SCM workers.

#### Perspective of system actors regarding the factors affecting supply of SCM workers

The results from system actors identified one theme affecting supply of SCM workers: 1) education, skills, and competencies.

1. Education, skills, and competencies

The majority of interviewees described how skills and supply issues vary across cadres. At health facilities, the health center level lacks permanent SCM staff; instead, nurses manage the pharmacy as an additional task. Nurses’ SCM skills are good; however, due to high workload, SCM is a lower priority. For mid-level SCM staff (e.g., warehouse manager, district pharmacist), hired workers have varied experiences, and often are grown within the system. Mid-level staff are experienced and have technical skills but few soft skills (e.g., leadership or management skills). For high-level SCM staff (e.g., directors, pharmacist-in-charge), workers are found mostly at the central and program levels. Staff at this level usually ascend through the ranks, growing through the pipeline. Across cadres, interviewees raised the importance that an adequate number of staff must exist and be trained in SCM.

*“At district pharmacies, people are trained [in SCM], but the numbers are not enough. At the hospital [level], there is only one pharmacist to manage supply chain and clinical pharmacist functions... I don’t think there is an issue of workforce at central level.”*

(System Actor Respondent 10)

*“Health center level doesn’t have permanent SCM staff. They have nurses that have to manage the pharmacy as an added task.”*

(System Actor Respondent 3)

### Perspectives from interviews with SCM demand-side functional actors

Twenty-two participants from 19 public institutions and private companies that employ SCM workers were interviewed (Table 2).

#### Perspective of employers on factors affecting demand for SCM workers

Results from SCM employers identified five themes about factors affecting organizational demand for SCM workers, including: 1) regulation, 2) education, skills, and competencies, 3) workload, 4) staff turnover, and 5) financial issues.

1. Regulation

Some interviewees expressed that regulation dictates the types of professionals required for SCM positions. Legislation requires that pharmacists manage pharmaceuticals, while clearance agents and the bonded warehouse environment in Rwanda are also under regulatory oversight for product’s integrity, safeguarding safety, and quality.

*“Often [needs are] driven by type of commodity. For example, pharmacists are sought to manage the drug supply chain. Lab [technicians] are sought to manage reagents or equipment, rather than [those with] supply chain qualification or expertise. This is somewhat policy driven. For example, a pharmacist needs to have overall responsibility for management of retail pharmacy. This is not true for supply chains for other commodities.”*

(Functional Actor: Demand Respondent 6)

2. Education, skills, and competencies

Some interviewees noted that SCM industries seek certain SCM skills depending on evolving needs. Interviewees reported difficulties obtaining mid-level SCM managers, suggesting this might be due to limited foundational schooling and the very low number of people trained in SCM.

*“[We] don’t have qualified people. The country has the standards but [we] don’t have the people for the standards. [There is] technical deficiency, lack of agility and lateral thinking.’’*

(Functional Actor: Demand Respondent 16)

*“[We] need a pharmacist as a technical person monitoring what is happening with pharmaceuticals. [We are] looking for skills for certain functions. [For example,] if interacting with customers, then nursing staff are needed.”*

(Functional Actor: Demand Respondent 17)

3. Workload

Some interviewees also expressed that as product volumes grow, workload grows and staffing needs increase. Interviewees raised concerns that this increased need for staff must align with budgetary provisions.

*“Increasing products or a higher amount of products [impacts demand].”*

(Functional Actor: Demand Respondent 16)

*“[There is] workload growth as volume grows.”*

(Functional Actor: Demand Respondent 17)

4. Staff turnover

Some interviewees raised the issue of staff being trained, then leaving the organization. Staff turnover increases the demand for new workers; however, organizational efforts to train the original employee are lost, discouraging investment in workforce development.

*“One of the struggles is you train people, and they leave. Last year, two people left after being with organization for two years.”*

(Functional Actor: Demand Respondent 3)

*“Any staff turnover [impacts demand].”*

(Functional Actor: Demand Respondent 17)

5. Financial issues

Some interviewees described how limited funding is allocated to SCM staff within the organization, which impacts the ability to sustain quality SCM activities, hire, and train staff.

*“Financial issues ([e.g.,] collection from clients, how soon are credits paid off, and [foreign exchange]issues) [affect demand].”*

(Functional Actor: Demand Respondent 11)

*“We have balanced procedure planning based on workload and available budget. We have been requesting an additional staff member, but budget is not great, so I’m not sure if we will get it.”*

(Functional Actor: Demand Respondent 1)

#### Perspective of employers on factors affecting the supply of SCM workers

Results from SCM employers identified four themes about factors influencing the supply of SCM workers, including: 1) education, skills, and competencies, 2) lack of career structure, 3) labor laws, and 4) outsourcing and networking.

1. Education, skills, and competencies

The majority of interviewees consistently described a lack of SCM education across all cadres and a lack of competencies and technical skills. Interviewees also cited a shortage of higher-level general skills, including critical thinking and problem solving, among applicants and workers. SCM curricula may not be aligned to required current workplace competencies.

Challenges of recruiting and hiring skilled staff vary markedly across career levels, with the biggest gap in the labor market existing for mid-level professionals. For technical positions, many applicants exist. For mid-level positions, many apply but few applicants have the required technical skills. Problem solving, integrity and initiative is often lacking. Upper-level candidates have adequate skills, with entrepreneurship and people management skills identified as more difficult-to-find. Predominantly, this level of SCM worker is available but sourced externally. Smaller organizations have more difficulty finding candidates.

*“Many people apply for jobs (or even when there are no open positions) – especially at mid- and lower-level. Very few have the level of skills and qualification sought – only about 15% would [we] consider interviewing.”*

(Functional Actor: Demand Respondent 3)

*“We hire people and do our own training – many do not have skills initially; we don’t have many skilled people for supply chain in Rwanda.”*

(Functional Actor: Demand Respondent 11)

*“We need more effort for logistics teaching in universities. Most employees have not acquired education. Their skills are from on-the-job [experience].”*

(Functional Actor: Demand Respondent 18)

2. Lack of career structure

Some interviewees noted the lack of a professional definition for health SCM and lack of career pathway in the system influences the supply of workers.

*“Supply chain ‘professional’ is not professionalized in Rwanda. [There’s] no career pathway. It isn’t clear where supply chain fits into organizational structures…*

*[We need a] career pathway in supply chain to delineate the various levels of the supply chain profession and how to progress between levels. Government should recognize how supply chain professionals are critical for the country’s economy and help to put it into the organizational structures of the various institutions.”*

(Functional Actor: Demand Respondent 6)

*“How can we…make sure people know ‘this is my job, my career’ and be professional and make a difference? These [factors] motivate and drive people. We are looking to certify people to bring them confidence and looking for professional identity and career path, a sense of mission to public health directives.”*

(Functional Actor: Demand Respondent 12)

3. Labor laws

Some interviewees indicated labor laws influence the movement of workers.

*“Rwanda labor law is very strict and does not bind workers to work over a certain period before leaving for better offers.”*

(Functional Actor: Demand Respondent 13)

4. Outsourcing and networking

Individual interviewees described how as the SCM sector grows, workers can find opportunities at other companies or competitors.

*“Can interact with other companies as part of the job and find other opportunities.”*

(Functional Actor: Demand Respondent 8)

### Perspectives from interviews with SCM supply-side functional actors

Three participants from three independent schools at the University of Rwanda listed in Table 2 were interviewed.

#### Perspective of universities about factors affecting demand for SCM workers

Results from SCM supply-side functional actors identified one theme about factors affecting organizational demand for SCM workers: 1) expanding local pharmaceutical industry.

1. Expanding local pharmaceutical industry

Some interviewees identified that the recent establishment of the Rwanda FDA and government plans to expand local pharmaceutical manufacturing could increase future demand for pharmacists with competency in regulatory affairs and SCM.

*“Rwanda FDA and the [expanding] local pharmacy industry may be increasing demand of pharmacists.”*

(Functional Actor: Supply Respondent 3)

#### Perspective of universities about factors affecting supply of SCM workers

Results from SCM supply-side functional actors identified five themes about factors influencing the supply of SCM workers, including: 1) limited SCM pharmacy curriculum, 2) disconnect between SCM industry and education sector, 3) facility and staff capacity, 4) lack of awareness of SCM career and courses, and 5) cost of education.

1. Limited SCM pharmacy curriculum

Interviewees from the School of Medicine and Pharmacy described how education for undergraduate pharmacy students focuses on entry-level competencies for all relevant domains; only one module covers SCM competencies. Graduating pharmacy students rely on employer’s staff orientation to prepare for SCM jobs.

*“It’s not possible to increase space for SCM curriculum to twelve months as [the curriculum] is too crowded… University can’t cater for all needs. Staff orientation and onboarding should prepare people for their job.”*

(Functional Actor: Supply Respondent 3)

2. Disconnect between SCM industry and education sector

The majority of interviewees described a disconnect between industry employing SCM workers and the academic institutions producing these workers. Interviewees indicated that industry seems unaware of what academia is offering and vice versa and identified that increased dialogue is needed.

*“Academia needs industry and industry needs us… [we] need to have full and frank discussion.”*

(Functional Actor: Supply Respondent 1)

3. Facility and staff capacity at educational institutions

The majority of interviewees observed that enrollment is limited by the small number of academic facilities and number of academic staff available at a university.

*“Student intake is limited by facilities and academic staff.”*

(Functional Actor: Supply Respondent 1)

*“Enrollments are controlled by facility and staff capacity. Demand is high so we have been increasing the number [of enrollees; however], there is more demand than supply.”*

(Functional Actor: Supply Respondent 3)

4. Lack of awareness of SCM career and courses

One interviewee described a lack of awareness of available SCM courses and of SCM-related career options among potential students as two issues influencing enrollment. High school students may not be aware of SCM career options.

5. Cost of education

One interviewee identified cost as a limiting factor for students and raised the need to consider the affordability of programs.

*“We need to consider the affordability of our programs.”*

(Functional Actor: Supply Respondent 1)

### Perspectives from the focus group

One focus group consisting of ten members was presented with two main seeding statements in poster format with a summary of the main themes consolidated from the interviews under those statements. Table 3 shows the themes presented to participants. Participants were then asked to verify if, in their experience, the summaries reflected the main factors under each statement or if key elements were missing.

**Table 3:** Theme summaries presented to focus group participants

*Factors that affect the availability or ‘supply’ of skilled SCM workers include:*
- Students’ ability to learn how to learn (primary, secondary, and tertiary education)
- High school student awareness of and interest in SCM as a career
- SCM credentialing (courses) available from professional institutions
- SCM academic courses (bachelor and master level) available from universities
- Teaching quality of SCM education (both teaching skills and materials)
- Curriculum content is relevant and aligned to needs of employers
- Workers have sufficient soft skills, including fast decision-making, problem-solving, entrepreneurship
- Graduates must have the practical skills needed to use their knowledge in the workplace
*Factors that affect the employment or ‘demand’ for SCM workers in organizations include:*
- Regulations (e.g., pharmacist for health products, clearance officers need registration)
- A growing economy, as more money spent meaning more products moved
- Increasing workload, which is related to: increased volume of existing products, increased range of products, and expanding to new sites and locations
- Restructure of the organization
- Staff leaving the organization for all reasons (staff turnover)
- Absence of a SCM career path
- Organizations must recognize the importance of supply chain and the need for skilled workers for SCM

#### Perspectives of focus group about factors affecting demand for SCM workers

Regarding factors affecting demand for SCM workers, the provided summary statements were accepted and five additions were proposed: 1) procurement officers are also regulated in Rwanda and may require professional accreditation depending on role; 2) both presence of and absence of career path affect demand for SCM workers; 3) regarding the need for organizations to recognize the importance of SCM workers, participants emphasized the need for organograms with defined SCM positions; 4) staff incentives should include mentorship, supervision, and feedback; 5) participants noted that Rwanda Public Procurement Agency could act as a type of council to provide standards and professional recognition for workers.

#### Perspectives of focus group about factors affecting supply of SCM workers

The provided summary statements about factors affecting supply of SCM workers were accepted and the following two additions were agreed by the group: 1) a need to develop managerial excellence in SCM staff to meet current demand for these skills and 2) a need for an increased focus on SCM refresher courses in the workplace to maintain and develop staff skills.

#### Perspectives of focus group about staff turnover

A third seeding question was provided to the group: ‘*Many organizations reported that staff turnover was low, but they had a problem retaining mid-level SCM workers. What can you add?*’ Within the general discussion, the following points were seen as important. 1) Participants believed that staff turnover is high but “horizontal promotion”, which occurs frequently within the health sector in general, contributes significantly to turnover. 2) The private sector was considered not stable, with respect to wages and longevity, compared with government. Participants noted that hospital SCM staff very often move due to lack of experience and hit a career ceiling very quickly. Staff move looking for growth and a positive work environment. Another difficulty experienced in the private sector occurred between pharmacist-in-charge and business owner, if professional practices and requirements conflicted with business practices or profit needs. 3) The main reasons for movement are for higher wages and increased experience, especially for more junior, mid-level career pharmacists and SCM managers.

### Interrelationship of results

As outlined in the background to this paper, labor markets for health SCM in Rwanda are a result of local supply and demand characteristics. These local characteristics have been revealed following interviews with system and functional actors (supply and demand) in this labor market and are presented above. The findings from all participant groups are synergistic, speaking to similar issues. No contradictions between participant groups were noted. Although themes have been described separately, there are interrelationships between identified themes and subthemes, beyond the obvious supply and demand interactions.

#### Interrelationship of themes as we consider the supply of SCM workers

The top issue identified regarding the supply of SCM workers was a skills mismatch between SCM workers’ skills and the competencies jobs require. Interviewees most frequently reported education, skills, and competencies of workers or applicants do not match needs. Several interconnected factors contribute to this misalignment: 1) lack of career structure for SCM, 2) a lack of professional definitions for health SCM, and 3) SCM curricula that do not match the needs of the workplace and the competencies required in the current market.

A lack of clear professional definitions affects the quality and relevance of education available for health SCM professionals. For example, graduates with a bachelor’s degree in pharmacy are expected to engage in significant SCM activities; however, the current curriculum doesn’t cater for developing detailed SCM competencies the labor market requires. In addition, interviewees described a disconnect and lack of coordination between the educational sector and industry in terms of curriculum content. Interviewees questioned whether current SCM education available in Rwanda for SCM degrees and health degrees containing SCM components reflected workplace needs.

This study revealed that the challenges of hiring skilled staff vary markedly across career levels, with the biggest gap existing at the mid-level professional level. In the public sector, a shortage of SCM competency among health workers exists for required SCM work; this shortage is particularly robust among cadres at health facilities. In the private sector, many companies report difficulty in obtaining mid-level SCM managers. Finding mid-level SCM workers with the required technical skills as well as higher-level general skills, such as critical thinking and problem-solving, is difficult.

#### Interrelationship of themes as we consider the demand for SCM workers

On the demand side, interviewees described high workload for SCM workers in both the public sector and private companies. At the same time, the level of workload is poorly understood and the numbers of SCM workers needed are poorly defined in the public and private sectors. SCM skills and competencies workers require are poorly defined. For example, job descriptions lack SCM content outlining skill requirements and minimum education requirements. Financial limitations and difficulty in approving funds for needed SCM positions hinder demand and the hiring of adequate numbers of health SCM workers in the public sector. In the private sector, difficulty approving funds for needed SCM positions also occurs, and staff turnover impacts the demand for new workers. These factors are widening the gaps between supply and demand in the SCM labor market in Rwanda. High staff turnover was a cross-cutting theme, affected by available wages and salaries, a lack of career path, and associated improvement of professional competence.

## Discussion

The research presented in this paper explored the perspectives of SCM-related employers, regulators, educational institutions, and professional associations regarding the factors influencing demand for and supply of SCM workers in Rwanda. Findings from this study indicate the SCM profession in Rwanda requires coordinated action from stakeholders, including the Ministry of Health in Rwanda, private supply chain companies, professional associations, educational institutions, policy makers, and development partners, to catalyze the SCM labor market. This study revealed that supply and skills of the health SCM workforce must be better aligned to match labor market demand. Reaching the required quantity and quality of the health SCM workforce requires that policy and funding choices about the SCM labor market and the education market are aligned with the needs of the country [39].

This study confirms and extends findings from previous research. A review exploring the root causes of underperformance in the health supply chains of LMICs identified a mismatch between staff skill and system design, as well as a lack of staff incentives, to be significant root causes of underperformance in public sector supply chains; in the private sector, the review identified a shortage of pharmacists and proliferation of informal drug sellers were significant causal factors of supply chain underperformance [40]. A study of the HSCM workforce in Rwanda identified some of the same challenges highlighted in this study, including high staff turnover among store managers, lack of dedicated staff to manage pharmacy, insufficient time for pharmacy activities given high workload, and limited training [13]. Studies of the health supply chain from Ethiopia, Uganda, Zambia, and Nigeria call out a range of workforce issues that were also revealed in this study: high staff mobility and lack of career structure (Ethiopia) [41], lack of governance and workforce capacity (Uganda) [42], higher job satisfaction in private sector (Zambia) [43], and a lack of SCM training and worker motivation (Nigeria) [44]. An earlier study exploring perspectives of health supply chain managers in Ghana [14] identified issues in line with the findings of this study: 1) SCM curricula at education institutions did not develop specific SCM skills, 2) insufficient SCM staff, and 3) increased workload. HSCM workers in Ghana [14] requested several competencies for strengthening, including in-depth training on SC functions, technical skills to understand regulatory and policy aspects of SCM, and managerial and customer care competencies – all competencies found to be lacking in the supply of SCM workers in Rwanda.

This study also includes new insights. Perspectives from the education sector identified several factors influencing the supply and production of SCM workers that will require attention, including limited facility and staff capacity at educational institutions, cost of education, lack of awareness of SCM courses, and a disconnect between academia and SCM industry.

With the WHO’s Roadmap for Access to Medicines, Vaccines and Health Product 2019–2023: Comprehensive Support for Access to Medicines, Vaccines and other Health Products supporting a workforce that is fit-for-purpose in key areas such as procurement and SCM [45], a systematic approach to developing the SCM workforce that considers the labor market will prove invaluable, especially as we consider the role of medicines and medical technologies in reaching the Sustainable Development Goals. Ensuring systematic investments beyond workforce training to address issues in the SCM of a country will also be important. Published in 2023, People that Deliver (PtD) conducted a business case study looking across budgeted investments for human resources for SCM by donors at a country level over the period from 2017–2020 [46]. The study revealed that significant investments have been made in staffing (wages and salaries) and training SCM staff, while broader workforce issues and enablers, including motivation and working conditions, have been neglected [46]. The study calls for increases in systematic workforce investments aligned to health product budget allocations [46].

The Global Fund invests US$4 billion a year to combat HIV, tuberculosis, and malaria, and to build sustainable health systems, with a large proportion of that funding invested in medical products and supply chain strengthening. Recognizing that issues in the SCM workforce could potentially risk its large investments in medical products and national health supply chains, the Global Fund has increasingly focused on systematic approaches to workforce development for the health SCM workforce and recently launched the Global Fund Supply Chain Governance and Workforce Development Framework to develop and professionalize capabilities in the SCM workforce and fill organizational capacity gaps [17]. This is one significant example of systematic supply chain strengthening for the health sector, which moves beyond a focus on training national SCM staff to address broader SCM workforce issues in LMICs.

### Recommendations related to supply issues relating to SCM workers in Rwanda

The authors provide various recommendations that support a systematic approach to developing the SCM workforce and can stimulate the health SCM labor market in Rwanda. In this study, employers interviewed identified the need for job-ready technical skills and higher-level problem-solving skills. Similar deficiencies have been noted in studies conducted in other LMICs [14, 42, 44]. These interviewees recommended education and training institutions provide more graduates with SCM skills required in the job market, noting that private sector organizations have a role to play with employee training and internships.

A career path for SCM workers that delineates the various levels of the SCM profession and how to progress between levels should be defined to encourage growth in the number and skill-level of SCM workers. A SCM professionalization framework that provides a standardized competency framework for SCM professionals would improve many of the issues interviewees described around the production of SCM workers by allowing the government to define standards for SCM professionals, employers to describe competency requirements, educational institutions to align SCM education to defined requirements, and SCM professionals to follow a career pathway and have a professional identity [4]. In particular, the government should consider defining health SCM pharmacists as a professional cadre or SCM as a recognized specialty for pharmacists, and link this to SCM professionalization framework discussions to create a career path with associated definitions for education and job roles. The authors are aware of similar efforts underway in Nigeria, Ghana, and Mozambique.

It is critical to consider the interrelationships between the health SCM job market and education market. Defined professional roles and the status of a profession can influence the number of and quality of applicants for education programs. Professional status and definitions also influence curriculum content [21, 47]. Once SCM professional standards are defined, educational institutions in Rwanda should apply a competency-based approach to review SCM curriculum needs for expected SCM cadres, while engaging SCM industry and other relevant stakeholders [4]. This process would foster the dialogue between industry and education sectors that is currently lacking.

Educational institutions must update and enrich their curriculum with content that will deliver the competencies demanded in the current market. Institutions should be encouraged to engage with public and private employers and consider partnering with other educational institutions within and outside the country to generate content to fill competency gaps. Also, a systematic comparison of curriculum content with leading global institutions of SCM may be helpful. The education sector could also periodically solicit feedback from employers on their students in industrial training or internship to gauge the relevance of their curriculum in the marketplace.

Interviewees identified other issues surrounding uptake of educational courses, which could influence the production of SCM workers in Rwanda. Course fees may prevent potential students from accessing courses; tuition fees can impact student demand for education, with higher fees relative to expected wages lowering student demand [21]. Lack of awareness of SCM careers among high school students also could impact enrollment in relevant programs. Lack of awareness among SCM workers and employers about existing SCM courses may cause underutilization of education offerings. This disconnect could prevent existing professionals from advancing their skills and progressing in their career and could be limiting the development of mid-career and higher-level professionals that interviewees reported are missing from the local workforce. Educational institutions should consider marketing strategies and financial policies that would promote industry awareness and uptake of SCM courses.

### Recommendations related to demand issues of SCM workers in Rwanda

MOH and private SCM organizations should conduct ‘Workload Indicators of Staffing Needs’ (WISN) workload analyses to determine requirements for numbers of SCM staff [48]. This method calculates the number of health workers needed per cadre, based on workload. The Government of Rwanda should also consider an advocacy campaign supported by SCM workload data to raise awareness and financial support for hiring public sector health SCM workers.

To improve SCM competencies, private companies and the MOH should consider conducting SCM Training Needs Analyses [49] and developing SCM capacity development plans that consider orientation, induction, and in-service training, as well as pre-service education. Public and private employers should review all job descriptions for cadres required to engage in SCM activities and clearly define SCM requirements.

The Government of Rwanda has established a strong policy framework and targets for economic development [20]. Other government plans, such as to increase the local pharmaceutical manufacturing industry, are expected to increase the demand for pharmacists. This study revealed significant concerns regarding the availability of skilled SCM human resources to match the government’s intentions to expand industry and improve health services access in Rwanda [20, 50]. Rwanda’s existing skills base is a constraint to growth of existing businesses (including supply chain and logistics), limits investment, and is not enabling a rapid transition to a middle-income economy [20].

Since this research was completed, the Rwanda MOH has begun to adapt the PtD SCM Professionalization Framework [4] to create a SCM professionalization framework to catalyze the labor market. The framework provides professional standards that align career path, education, and professional growth in health supply chain management. At its core is the Library of Competencies and Designations, which provides standards to ensure alignment between the supply of and demand for health supply chain professionals [4]. This framework also contains an implementation approach, which provides clear guidance on how to begin this journey of change [4].

### Limitations

Stakeholders interviewed might not represent the views of all experts involved in the SCM labor market. Although the interviews conducted represented the main stakeholders related to the research question, it is possible that a greater number of interviews across these stakeholders may have yielded different results. In the same way, although all interviewees were invited to participate in the focus group, not all attended. As such, the validation exercises conducted may have yielded varying results with different or more participants. As the scope was limited to the City of Kigali, regional and remote perspectives are not represented. Factors affecting supply and demand of the SCM workforce might be more pronounced in regional and remote areas. While we believe the main findings would not be significantly altered by further inputs, results could be strengthened by the voices of certain system actors (i.e., Ministry of Education, RPPA, RDB), supply actors (i.e., private universities), and other demand actors, including pharmaceutical manufacturing companies, BUFMAR (which distributes pharmaceutical products to faith-based health facilities), and additional private companies involved in non-pharmaceutical supply chains. In addition, interviews and focus groups were conducted in English. While English is one of the four official languages in Rwanda, some participants might have had higher proficiency in another official language. Future studies in this area would benefit from exploring the opinions of HSCM workers, students, or recent graduates.

## Conclusions

This study adds to the current understanding of factors influencing the SCM labor market in Rwanda by documenting perspectives from system actors, universities, and employers of SCM workers. This study revealed gaps between the skills and competencies of the supply of SCM workers in Rwanda and the competencies SCM jobs require. Improving the SCM labor market in Rwanda and the availability of the skilled cadres needed for the ongoing, effective management of health supply chains requires coordinated efforts by the Ministry of Health in Rwanda, private SCM companies, professional associations, education sector, and policy makers. National stakeholders in other country contexts can adapt this methodology to identify factors driving the supply of and demand for SCM labor and develop effective policies to address labor market imbalances.

### Supplementary Information

**Additional file 1.** Targeted Literature Review. (Table: Targeted review of the literature on health and SCM labor market issues).

**Additional file 2.** Study Interview Guide.

## Data Availability

The datasets used and/or analyzed during the current study are available from the corresponding author on reasonable request.

## Abbreviations

BSc: Bachelor of Science
BUFMAR: Le Bureau des Formations médicales agréées du Rwanda
FDA: Food and Drugs Authority
GHSC-PSM: Global Health Supply Chain Program–Procurement and Supply Management
HR: Human resources
LMIC: Low- or middle- income country
MAGERWA: Magasins généraux du Rwanda
MINICOM: Ministry of Trade and Industry
MOH: Ministry of Health
MPPD: Medical Procurement and Production Division
NGO: Nongovernmental organization
PtD: People that Deliver
RCE-VIHSCM: Regional Centre of Excellence for Vaccine Immunization and Health Supply Chain Management
RDB: Rwanda Development Board
RPPA: Rwanda Public Procurement Agency
SCM: Supply chain management
SDG: Sustainable Development Goal
UHC: Universal health coverage
UNHCR: United Nations High Commissioner for Refugees
USAID: United States Agency for International Development
WISN: Workload Indicators of Staffing Needs
