# Supplementary material for: Labor markets for health supply chain management in Rwanda: a qualitative study of stakeholder perspectives
Source: BMC Health Serv Res. 2023 Dec 7;23:1376. doi: 10.1186/s12913-023-10304-1 (PMC10704744; doi:10.1186/s12913-023-10304-1)
Supplement: Supplementary file 1 — Additional file 1. Targeted Literature Review. (Table: Targeted review of the literature on health and SCM labor market issues). [file 12913_2023_10304_MOESM1_ESM.docx]

**Additional File 1. Targeted review of the literature on health and SCM labor market issues**

| **Authors & Year** | **Country** | **Purpose** | **Sector** | **Target Population** | **Key Findings** |
| --- | --- | --- | --- | --- | --- |
| Karan A, Negandhi H, Kabeer M, Zapata T, Mairembam D, De Graeve H, et al. 2023 | India | To estimate actions and funding needed to increase the production of health workers to achieve UHC | Health | Doctors, nurses, midwives | India must create new medical colleges to increase production of doctors, nurses, and midwives to achieve UHC.  Raising demand for workers in the health system, improving working conditions, and filling existing vacancies will also be needed. |
| Al Harrasi N, Salah El Din M, Reason M, Al Balushi B, Al Habsi J. 2023 | Oman | To explore managers’ perceptions of 1) the importance of knowledge/skills in the Logistics and SCM degree and 2) knowledge/skills gaps of entry-level graduates | Supply Chain Management | Entry-level Logistics and SCM graduates | Managers’ satisfaction level of entry-level logistics professionals’ knowledge and skills was below expectations. Moderate gaps existed in graduates’ knowledge and skills. |
| Nuruzzaman M, Zapata T, McIsaac M, Wangmo S, Islam MJ, Almamun M, et al. 2022 | Bangladesh | To provide an overview of 1) production of health workers, 2) supply and demand in the public sector, and 3) supply in the private sector | Health | Health workforce (e.g., doctors, nurses and midwives, dentists, pharmacists, others) | Density of doctors, nurses and midwives was well below the Sustainable Development Goals (SDG) index threshold.  Processes to regulate and collect data on health worker supply and demand will be required.  Although health worker education institutions have increased, graduates’ skill-mix and training quality must be considered. |
| Garg S, Tripathi N, McIsaac M, Zurn P, Zapata T, Mairembam DS, et al. 2022 | India | To investigate the extent to which recommendations of a previous Health Labor Market Analysis were implemented and whether HRH availability improved. | Health | Doctors, specialist doctors, nurses, community health officers | Uneven availability of doctors and other health workers existed with severe shortages in rural areas.  Substantial production of nurses occurs, particularly from private schools, however trusted accreditation mechanism was lacking.  Vacancies in public sector persisted alongside nurse unemployment. |
| Okoroafor SC, Kwesiga B, Ogato J, Gura Z, Gondi J, Jumba N, et al. 2022 | Kenya | To understand how health work force supply, demand, and needs relate to each other | Health | Nurses, clinical officers, and doctors | 32% gap existed in the density of doctors, nurses and clinical workers to reach the SDG index threshold.  Kenya must align production of health workers to population needs. |
| Asamani JA, Zurn P, Pitso P, Mothebe M, Moalosi N, Malieaneet T, et al. 2022 | Lesotho | To quantify the needs, demand, supply in the health labor market and financial space requirements to meet needs | Health | Health workers across 18 health occupations | 53% gap identified between health worker stock and population needs.  Unemployment of some cadres persisted alongside this gap.  Health workforce budget must increase by at least 12.3% each year to address gaps. |
| Ministry of Health, Government of Rwanda. 2019 | Rwanda | To assess human resources for health situation in Rwanda, including demand factors and supply factors, in the public and private health sector | Health | All clinical cadres (e.g., doctors, nurses, midwives, allied health professionals, pharmacists, etc.) | Despite growth in number of nurses, midwives and pharmacists, future projections indicate a shortage of some health workers, where level of production does not meet projected needs. |
| Sinha A, Millhiser WP, He Y. 2016 | United States | To investigate gaps in demand for and supply of SCM-related knowledge areas | Supply Chain Management | Business school SCM curricula and employer job requirements | Gaps exist between the SCM talent requirements described by industry and the knowledge/skill training by US business schools. |
| Sousa A, Scheffler RM, Koyi G, Ngah SN, Abu-Agla A, M’kiambati HM, et al. 2014 | Cameroon, Kenya, Sudan, and Zambia | To assess key indicators of the health labor market dynamics and to identify the policies implemented to address workforce shortages | Health | Doctors, nurses, midwives, others | Major shortages and maldistribution in health workers availability existed in all four countries. Factors including migration, aging workforce, and imbalances in existing skill mix exacerbate these problems. |
| Yew Wong C, Grant DB, Allan B, Jasiuvian I. 2014 | United Kingdom | To examine the curriculum design of logistics and SCM undergraduate courses at selected higher education institutions and compare them with job requirements described by employers | Supply Chain Management | Logistics and SCM undergraduate curriculum and employer job requirements | Imbalance exists between undergraduate curriculum and employer needs. |
| Sousa A, Scheffler RM, Nyoni J, Boerma T. 2013 | Global | To present a health  labor market framework that can provide a comprehensive approach needed to understand the factors influencing supply and demand in the health workforce | Health | Health workforce | Health workforce policies like those that focus on education are partial, not comprehensive, and are not effective in addressing health workforce shortages. Conditions in the health labor market must exist that also enable health workers and graduates to be taken into the health workforce. |
