# Supplementary material for: Labor markets for health supply chain management in Rwanda: a qualitative study of stakeholder perspectives
Source: BMC Health Serv Res. 2023 Dec 7;23:1376. doi: 10.1186/s12913-023-10304-1 (PMC10704744; doi:10.1186/s12913-023-10304-1)
Supplement: Supplementary file 2 — Additional file 2. Study Interview Guide. [file 12913_2023_10304_MOESM2_ESM.docx]

**Additional File 2 – Study Interview Guide**

**Structured interview questions for key informant interviews**

| 1. **INTRODUCTIONS** |
| --- |

| 1. **BACKGROUND** | | | | | | | | | |
| --- | --- | --- | --- | --- | --- | --- | --- | --- | --- |
| - 1. **Name of respondent** | | | | | | | | | |
|  | | | | | | | | | |
| - 1. **Name of organization** | | | | | | | | | |
|  | | | | | | | | | |
| - 1. **Data collector (name and organization)** | | | | | | | | | |
|  | | | | | | | | | |
| - 1. **Date** | | | | - 1. **Start time** | | | | | |
|  | | | |  | | | | | |
| - 1. **Administrative region** | | | | | | | | | |
| Kigali | | | | | | | | | |
| - 1. **District** | | | | | | | | | |
|  | | | | | | | | | |
| - 1. **Type of setting (Highlight in yellow to select response)** | | | | | | | | | |
| Government secto**r** | | | International Development sector | | | | | Academic sector | |
| Humanitarian sector | | | Private sector | | | | | Other. Please specify: | |
| - 1. **Type of organization (Highlight in yellow to select response)** | | | | | | | | | |
| Federal government | | | | | | Bilateral donor (e.g. USAID, DFID) | | | |
| Regional government | | | | | | Technical Partner/NGO (e.g. Chemonics, Oxfam, John Snow Incorporated, Save the Children, VillageReach etc.) | | | |
| 3^rd^ Party Logistics Provider (3PL) (e.g. DHL, UPS) | | | | | | Private sector company (e.g. Mining company, goods wholesaler etc.) | | | |
| 4^th^ Party Logistics Provider (4PL) (e.g. Government health center) | | | | | | Other. Please specify: | | | |
| United Nations Agency (e.g. WHO, UNICEF, UNFPA, WFP**)** | | | | | |  | | | |
| - 1. **Type of products your organization supports the movement of (Highlight in yellow to select response)** | | | | | | | | | |
| Health products | | | Household goods | | | | | Other. Please specify: | |
| Food | | | Mining and resources | | | | | N/A | |
| 1. **PERSONAL BACKGROUND** | | | | | | | | | |
| - 1. **Gender (Highlight in yellow to select response)** | | | | | | | | | |
| Male | | | | | | Female | | | |
| - 1. **Year of Birth** | | | | | | | | | |
|  | | | | | | | | | |
| - 1. **Nationality (Highlight in yellow to select response)** | | | | | | | | | |
| Rwandan | | | Expatriate | | | | | Other. Please specify: | |
| - 1. **Title of Position** | | | | | | | | | |
|  | | | | | | | | | |
| 1. **PROFESSIONAL EXPERIENCE** | | | | | | | | | |
| - 1. **What is your current position in this facility? (Highlight in yellow to select response)** | | | | | | | | | |
| Health professional. Please specify: | | | | | | | | | |
| Logistics or Supply Chain professional. Please specify: | | | | | | | | | |
| Other. Please specify: | | | | | | | | | |
| - 1. **How long have you been in this position? (Highlight in yellow to select response)** | | | | | | | | | |
| Less than 6 months | | | | | | 10 years or more | | | |
| Between 6 months and less than 12 months | | | | | | Do not know/remember | | | |
| Between 1 year and less than 5 years | | | | | | Not answered | | | |
| Between 5 years and less than 10 years | | | | | |  | | | |
| 1. **ORGANIZATION STAFF PROFILE** | | | | | | | | | |
| ***Can you please provide us with the following details regarding the staff profile of your organization?*** | | | | | | | | |  |
| - 1. **How many dedicated SCM or logistics staff positions does your organization have at a mid- or upper-level management level?** | | | | | | | | |  |
| Full Time | | Part Time | | | | | Consultants and “Casual Employees” | |  |
|  | |  | | | | |  | |  |
| - - 1. **Of these mid- and upper-level management positions, what percentage is female?** | | | | | | | | |  |
|  | | | | | | | | |  |
| - 1. **How many dedicated SCM or logistics staff positions does your organization have at a technical level (e.g. lift drivers, packers etc.)?** | | | | | | | | |  |
| Full Time | | Part Time | | | | | Consultants and “Casual Employees” | |  |
|  | |  | | | | |  | |  |
| - - 1. **Of these technical-level SCM and logistics positions, what percentage is female?** | | | | | | | | |  |
|  | | | | | | | | |  |
| - 1. **How many NEW SCM and logistics positions do you expect your organization to offer in the coming 1yr and 5 yrs.?** | | | | | | | | |  |
|  | | | | | | | | |  |
| - 1. **How many current SCM and logistics staff do you feel will leave the organization in the coming 1yr and 5 yrs.?** | | | | | | | | |  |
|  | | | | | | | | |  |
| 1. **SUPPLY AND DEMAND** | | | | | | | | | |
| - 1. **In your experience what are the factors that affect your organization’s demand for SCM workers?** | | | | | | | | |  |
|  | | | | | | | | |  |
| - 1. **What would increase your organization’s demand for more SCM workers at various skills levels?** | | | | | | | | |  |
|  | | | | | | | | |  |
| - 1. **In your experience what are the factors that affect the current availability of skilled SCM workers in the labor market in Rwanda?** | | | | | | | | | |
|  | | | | | | | | | |
| - - 1. **Are there specific factors that affect the availability of *technical SCM and logistics staff*?** | | | | | | | | | |
|  | | | | | | | | | |
| - - 1. **Are there specific factors that affect the availability of *mid-level SCM and logistics managers*?** | | | | | | | | | |
|  | | | | | | | | | |
| - - 1. **Are there specific factors that affect the availability of *upper-level SCM and logistics managers*?** | | | | | | | | | |
|  | | | | | | | | | |
| - 1. **How do you think the supply of SCM workers could be improved?** | | | | | | | | |  |
|  | | | | | | | | |  |
| 1. **HIRING PRACTICES** | | | | | | | | | |
| - 1. **What qualifications and experience do you look for when seeking to hire SCM technical Staff?** (Breakdown by type) | | | | | | | | |  |
| **TYPE OF STAFF** | | | | | **REQUIREMENT** | | | |  |
|  | | | | |  | | | |  |
|  | | | | |  | | | |  |
|  | | | | |  | | | |  |
| **Comments**: | | | | | | | | |  |
| - 1. **What qualifications and experience do you look for when seeking to employ SCM mid-level and upper level managers?** (breakdown by type) | | | | | | | | |  |
| **TYPE OF STAFF** | | | | | **REQUIREMENT** | | | |  |
|  | | | | |  | | | |  |
|  | | | | |  | | | |  |
|  | | | | |  | | | |  |
| **Comments**: | | | | | | | | |  |
| - 1. **Please provide an overview of your recruitment and hiring process?** | | | | | | | | | |
|  | | | | | | | | | |
| 1. **BENEFITS** | | | | | | | | | |
| - 1. **Would you be comfortable to share with us general salary ranges for SCM technical staff, mid-level managers and upper-level managers to allow us to compare to other SCM industry sectors?** | | | | | | | | |  |
| **TYPE OF STAFF** | | | | | **WAGE RANGE** | | | |  |
| Technical-Level | | | | |  | | | |  |
| Mid-Level Management | | | | |  | | | |  |
| Upper-Level Management | | | | |  | | | |  |
| **Comments**: | | | | | | | | |  |
| - 1. **Please provide an overview of the benefits package you offer your employees, with consideration to financial and non-financial rewards.** | | | | | | | | |  |
| **TYPE OF STAFF** | | | | | **BENEFIT PACKAGE** | | | |  |
| Technical-Level | | | | |  | | | |  |
| Mid-Level Management | | | | |  | | | |  |
| Upper-Level Management | | | | |  | | | |  |
| **Comments**: | | | | | | | | |  |
| 1. **SCOPE OF WORK** | | | | | | | | | |
| - 1. **Please provide an overview of the workload expectations (in terms of hrs./week) of SCM employees?** | | | | | | | | |  |
|  | | | | | | | | |  |
| - 1. **Do you provide job descriptions for your employees and how are these created?** | | | | | | | | |  |
|  | | | | | | | | |  |
| 1. **SATISFACTION WITH EMPLOYEES** | | | | | | | | | |
| - 1. **How satisfied are you with the skill level of TECHNICAL SCM EMPLOYEES when they start work with you? (Highlight in yellow to select response)** | | | | | | | | | |
| Very satisfied | | | Satisfied | | | | | Neutral | |
| Unsatisfied | | | Very unsatisfied | | | | | Not answered | |
| - 1. **How satisfied are you with the skill level of MID-LEVEL SCM MANAGEMENT when they start work with you? (Highlight in yellow to select response)** | | | | | | | | | |
| Very satisfied | | | Satisfied | | | | | Neutral | |
| Unsatisfied | | | Very unsatisfied | | | | | Not answered | |
| - 1. **How satisfied are you with the skill level of UPPER-LEVEL SCM MANAGEMENT when they start work with you? (Highlight in yellow to select response)** | | | | | | | | | |
| Very satisfied | | | Satisfied | | | | | Neutral | |
| Unsatisfied | | | Very unsatisfied | | | | | Not answered | |
| - 1. **Please elaborate on any concerns you have** | | | | | | | | | |
|  | | | | | | | | | |
| 1. **RETENTION** | | | | | | | | | |
| - 1. **What is your annual staff turnover rate for** | | | | | | | | | |
| **TYPE OF STAFF** | | | | | | **STAFF TURNOVER RATE AS A PERCENTAGE** | | | |
| Technical staff | | | | | |  | | | |
| Mid-level mangers | | | | | |  | | | |
| Upper-level managers | | | | | |  | | | |
| **Comments**: | | | | | | | | | |
| - 1. **What comments would you like to make regarding staff retention? How do you project and plan for staff attrition?** | | | | | | | | |  |
|  | | | | | | | | |  |
| 1. **IN-SERVICE TRAINING** | | | | | | | | | |
| - 1. **Please provide an overview of your staff orientation, mentoring and in-service training programs.** | | | | | | | | |  |
| **Staff orientation** |  | | | | | | | |  |
| **Mentoring** |  | | | | | | | |  |
| **In-Service training** |  | | | | | | | |  |
| **Comments**: | | | | | | | | |  |
| 1. **SUPPORTIVE SUPERVISION AND PERFORMANCE EVALUATION** | | | | | | | | | |
| - 1. **Please provide an overview of your supportive supervision and performance evaluation procedures.** | | | | | | | | | |
|  | | | | | | | | | |
| 1. **CONCLUSION** | | | | | | | | | |
| - 1. **What other comments would you like to make?** | | | | | | | | | |
|  | | | | | | | | | |
| - 1. **Interview end time** | | | | | | | | | |
|  | | | | | | | | | |
